# Factors Predicting Engagement of Older Adults With a Coach-Supported eHealth Intervention Promoting Lifestyle Change and Associations Between Engagement and Changes in Cardiovascular and Dementia Risk: Secondary Analysis of an 18-Month Multinational Randomized Controlled Trial

**DOI:** 10.2196/32006

**Published:** 2022-05-09

**Authors:** Nicola Coley, Laurine Andre, Marieke P Hoevenaar-Blom, Tiia Ngandu, Cathrien Beishuizen, Mariagnese Barbera, Lennard van Wanrooij, Miia Kivipelto, Hilkka Soininen, Willem van Gool, Carol Brayne, Eric Moll van Charante, Edo Richard, Sandrine Andrieu, HATICE study group, PRODEMOS study group

**Affiliations:** 1 Center for Epidemiology and Research in Population health (CERPOP) University of Toulouse III Paul Sabatier (UPS), National Institute of Health and Medical Research (INSERM) mixed research unit (UMR) 1295 Toulouse France; 2 Department of Epidemiology and Public Health Toulouse University Hospital Toulouse France; 3 Department of Neurology Donders Institute for Brain, Cognition and Behaviour Radboud University Medical Centre Nijmegen Netherlands; 4 Department of Neurology Amsterdam University Medical Center University of Amsterdam Amsterdam Netherlands; 5 Department of Public and Occupational Health Amsterdam University Medical Center University of Amsterdam Amsterdam Netherlands; 6 Population Health Unit Finnish Institute for Health and Welfare Helsinki Finland; 7 Division of Clinical Geriatrics, Center for Alzheimer Research Care Sciences and Society (NVS) Karolinska Institutet Stockholm Sweden; 8 Department of General Practice Amsterdam UMC University of Amsterdam Amsterdam Netherlands; 9 Institute of Clinical Medicine Department of Neurology University of Eastern Finland Kuopio Finland; 10 Ageing Epidemiology Research Unit School of Public Health Imperial College London London United Kingdom; 11 Institute of Public Health and Clinical Nutrition University of Eastern Finland Kuopio Finland; 12 Neurocenter Neurology Kuopio University Hospital Kuopio Finland; 13 Cambridge Public Health University of Cambridge Cambridge United Kingdom; 14 See Acknowledgments

**Keywords:** aging, eHealth, disparities, engagement, prevention, cardiovascular, lifestyle, risk factors

## Abstract

**Background:**

Digital health interventions could help to prevent age-related diseases, but little is known about how older adults engage with such interventions, especially in the long term, or whether engagement is associated with changes in clinical, behavioral, or biological outcomes in this population. Disparities in engagement levels with digital health interventions may exist among older people and be associated with health inequalities.

**Objective:**

This study aimed to describe older adults’ engagement with an eHealth intervention, identify factors associated with engagement, and examine associations between engagement and changes in cardiovascular and dementia risk factors (blood pressure, cholesterol, BMI, physical activity, diet, and cardiovascular and dementia risk scores).

**Methods:**

This was a secondary analysis of the 18-month randomized controlled Healthy Ageing Through Internet Counselling in the Elderly trial of a tailored internet-based intervention encouraging behavior changes, with remote support from a lifestyle coach, to reduce cardiovascular and cognitive decline risk in 2724 individuals aged ≥65 years, recruited offline in the Netherlands, Finland, and France. Engagement was assessed via log-in frequency, number of lifestyle goals set, measurements entered and messages sent to coaches, and percentage of education materials read. Clinical and biological data were collected during in-person visits at baseline and 18 months. Lifestyle data were self-reported on a web-based platform.

**Results:**

Of the 1389 intervention group participants, 1194 (85.96%) sent at least one message. They logged in a median of 29 times, and set a median of 1 goal. Higher engagement was associated with significantly greater improvement in biological and behavioral risk factors, with evidence of a dose-response effect. Compared with the control group, the adjusted mean difference (95% CI) in 18-month change in the primary outcome, a composite z-score comprising blood pressure, BMI, and cholesterol, was −0.08 (−0.12 to −0.03), −0.04 (−0.08 to 0.00), and 0.00 (−0.08 to 0.08) in the high, moderate, and low engagement groups, respectively. Low engagers showed no improvement in any outcome measures compared with the control group. Participants not using a computer regularly before the study engaged much less with the intervention than those using a computer up to 7 (adjusted odds ratio 5.39, 95% CI 2.66-10.95) or ≥7 hours per week (adjusted odds ratio 6.58, 95% CI 3.21-13.49). Those already working on or with short-term plans for lifestyle improvement at baseline, and with better cognition, engaged more.

**Conclusions:**

Greater engagement with an eHealth lifestyle intervention was associated with greater improvement in risk factors in older adults. However, those with limited computer experience, who tended to have a lower level of education, or who had poorer cognition engaged less. Additional support or forms of intervention delivery for such individuals could help minimize potential health inequalities associated with the use of digital health interventions in older people.

## Introduction

### Background

The number of people aged ≥60 years increased from 382 million worldwide in 1980 to 962 million in 2017 and is expected to reach nearly 2.1 billion by 2050 [1]. In parallel, there are an increasing number of cases of age-related diseases, including cardiovascular disease (CVD) and dementia, placing an ever-increasing burden on health and social care systems [2]. For example, the worldwide cost of dementia increased by 35% between 2010 and 2015 to reach US $818 billion and was estimated to exceed US $1 trillion in 2018 [3]. CVD and dementia share many potentially modifiable lifestyle-based risk factors, including physical inactivity, unhealthy diet, obesity, and hypertension [4,5], offering opportunities for prevention that could bring about huge public health gains. There is a need to establish, using rigorously conducted research studies, the extent to which interventions might influence behavior in the short, medium, and longer term, providing evidence for the best policies to reduce noncommunicable disease risk.

Digital health tools are a possible approach for delivering preventive interventions for CVD and dementia [6,7], which, if effective and efficient, can be rolled out at scale. However, nonuse of digital health interventions is a fundamental problem, with persistent reports of high discontinuation rates, even in research studies involving atypically motivated individuals [8,9]. Specifying the *dose* of nonpharmacological interventions, in terms of engagement, is inherently more difficult than for drug treatments, even more so for digital interventions where use is often at participants’ discretion [8] and requires more investment and motivation than simply taking a daily medication [10]. Although increased engagement with digital interventions is associated with greater improvements in health outcomes, including behavior change, in young and middle-aged adults [10-12], very little is known about engagement with digital interventions [13], or its association with health outcomes, in older people, especially in the long term.

A concern about using digital health interventions in older populations is that they may further widen existing health inequalities [14]. Although the use of digital technologies is increasing in this age group, *digital exclusion* is still common, particularly in individuals aged >75 years, and older adults who do not use the internet have poorer health and lower socioeconomic status than those who do [15]. Even among older internet users, the levels of engagement with digital technologies may vary and be associated with individuals’ characteristics. It is vital to understand better how older people use and interact with such tools and to identify potential disparities in use.

### Objectives

To explore this, we drew on data from a large international trial of an eHealth intervention designed to encourage behavior changes for the prevention of CVD and cognitive decline in older individuals to (1) describe engagement with the different components of the eHealth intervention, (2) identify factors associated with engagement, and (3) examine associations between engagement and changes in cardiovascular and dementia risk factors.

## Methods

### Setting and Participants

We analyzed data from the previously described 18-month Healthy Ageing Through Internet Counselling in the Elderly (HATICE) parallel group randomized controlled trial (ISRCTN48151589) [16-18]. Between March 2015 and August 2016, a total of 2724 dementia-free community dwellers aged ≥65 years with at least basic computer literacy and either 2 or more CVD risk factors (hypertension, dyslipidemia, overweight, smoking, or physical inactivity) or a history of CVD or diabetes were enrolled in Finland, France, and the Netherlands. Participants were randomized in a 1:1 ratio to either (1) the intervention, a multicomponent internet-based platform designed to encourage lifestyle changes, with remote support from a lifestyle coach (see the *Intervention* section), or (2) a control group receiving access to a simple static internet platform containing only basic health information and no coach support. Participants were recruited offline, primarily through a population registry (Finland), commercial mailing lists and a prevention center (France), and general practitioners (Netherlands) [17,19]. The intervention had a modest but significant beneficial effect on the trial’s primary outcome, a composite cardiovascular risk score [17]. Clinical, demographic, and biological data were collected during face-to-face study visits at baseline and 18 months. Data concerning lifestyle, mood, and health self-management were self-reported via the study’s web-based platform at baseline and 12 and 24 months. Adverse events were self-reported on the web-based platform every 3 months.

### Ethics Approval

The local ethical committees in each country approved the protocol (Academic Medical Centre, the Netherlands: METC 2014_126; Northern Savonia Hospital District Research Ethics Committee, Finland: 35/2014; Comité de Protection des Personnes Sud Ouest et Outre Mer, France: 2014-A01287–40), and all participants provided written informed consent.

### Intervention

**Figure 1:**
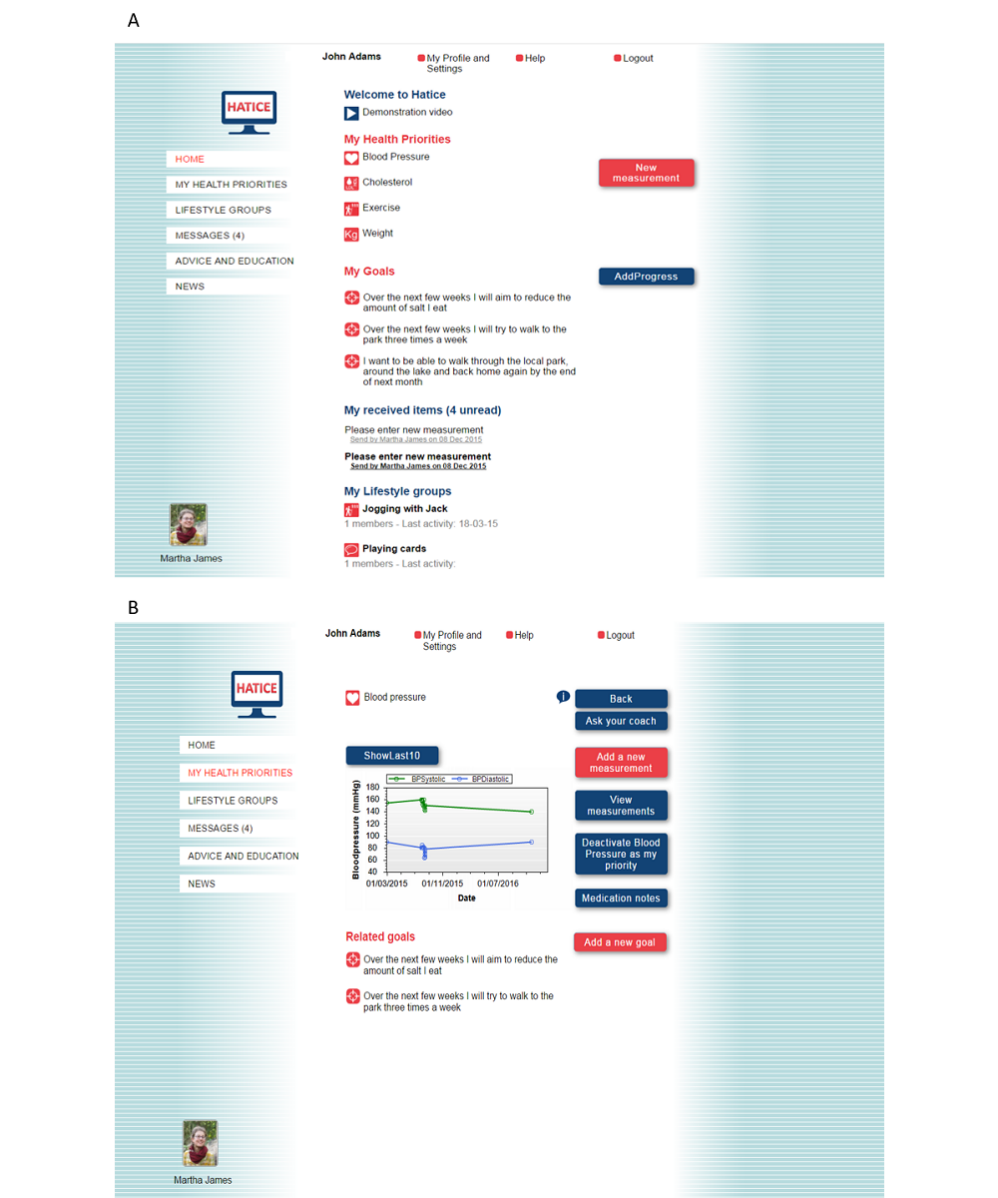
Screenshots of the HATICE intervention platform: (A) home page and (B) measurements page. HATICE: Healthy Ageing Through Internet Counselling in the Elderly.

Intervention group participants had access to a secure internet-based platform (Figure 1), with remote support from a lifestyle coach trained in motivational interviewing and healthy lifestyle advice. Full details of the development and content of the platform have been previously published [18]. The intervention aimed to facilitate the self-management of cardiovascular risk factors, including hypertension, obesity, physical inactivity, diet, smoking, diabetes, and hypercholesterolemia, to improve the overall risk profile. It was designed using national and European guidelines for primary and secondary CVD prevention [20] and input from members of the target population, health professionals, and patient organizations [16,21,22].

After secure login, participants were able to (1) view their individual cardiovascular risk profile (based on baseline measurements), (2) set personal goals for lifestyle change and make corresponding action plans, (3) monitor goals by entering data (eg, blood pressure measurements or food diaries) and receive graphical or automated feedback, and (4) obtain health information from education modules (including text, videos, and quizzes) and peer-to-peer videos. News items related to CVD, healthy aging, or eHealth were added regularly to the platform. All content was provided in the local language, and advice was adapted to local guidelines where necessary. Owing to the older age of the study’s participants and their expected level of computer experience, the navigation structure and layout of the intervention platform were kept as simple as possible (Figure 1).

Coaches met with participants face-to-face at baseline and thereafter communicated with them via a computer messaging system. There was also a booster telephone call at 12 months. Using motivational interviewing techniques, they supported participants in making lifestyle changes by encouraging them to prioritize up to 3 health factors (the home page layout then reflected the chosen health priorities) and set at least one goal at baseline, interact with the platform, and set additional goals over time. Coaches also provided motivational feedback. Participants could see a photograph and the name of their coach when they logged in to the intervention platform (Figure 1).

The intervention was designed by the academic researchers involved in the project. Technical development was performed by Vital Health Software.

### Outcomes

#### Engagement Outcomes

Engagement with digital health interventions, similar to engagement with serious games [23], is thought to encompass behavioral, emotional, and cognitive factors [24-26], but there is no consensus on how it should be measured [10]. Similar to previous studies [10,11,27,28], we assessed engagement (only in the intervention group) with our eHealth intervention through system use metrics, including number and dates of logins, number of goals set, messages sent to coaches, monitoring measurements or goal diary entries, and percentage of advice and education materials read.

Different studies have found different components of eHealth intervention use, including number of logins, number of activities completed per login, percentage of study modules completed, amount of goals set, and number of self-monitoring measures, to be associated with outcomes [26,29,30], and it has been suggested that composite measures may be the best way to measure engagement or adherence with such interventions [10]. Therefore, we developed a composite indicator of overall engagement throughout the study, defined as the sum of the points obtained for logins (0, 1, and 2 points for the first, second, and third tertiles of total logins, respectively), number of goals set (0: 0 points; 1: 1 point; ≥2: 2 points), sending of at least one message to the coach (no: 0 points; yes: 1 point), entering at least one measurement (no: 0 points; yes: 1 point), and reading some of the advice and education materials (no: 0 points; yes: 1 point). The composite engagement score ranged from 0 to 7 points, with higher scores indicating greater engagement. It was categorized into low (0-2 points), moderate (3-5 points), and high (6-7 points) engagement, thus avoiding potential difficulties with nonlinear relationships with outcome measures [26,29]. This categorization was decided before the analysis and was intended to capture *meaningful* differences in engagement with the platform use among the 3 groups (ie, *low*, *moderate*, and *high* engagers). In sensitivity analyses, we categorized the engagement scores using tertiles.

In addition, login data were used to study platform use over time. As participants were asked to log in every 3 months to complete an adverse event questionnaire, and at 12 and 18 months to complete study evaluations, and we could not distinguish these logins from other types, rather than calculating the time to last login, we calculated the time to the first occurrence of nonuse attrition, that is, no login during the previous month [8]. Because they only logged in once every 3 months, at most, participants who only logged in to complete adverse event questionnaires or study evaluations (and did not otherwise use the intervention platform) were considered to display nonuse attrition.

#### Risk Factor Outcomes

The trial’s primary outcome was a composite cardiovascular risk *z* score based on systolic blood pressure, low-density lipoprotein cholesterol, and BMI [17]. Secondary outcomes included individual components of the composite *z* score, physical activity [31], dietary intake (Mediterranean Diet Adherence Screener score) [32], and estimated cardiovascular (Systematic Coronary Risk Estimation–Older People) [33] and dementia (Cardiovascular Risk Factors, Aging, and Incidence of Dementia score) [34] risk.

#### Predictor Variables

The following baseline variables were assessed as predictors of engagement and nonuse attrition: age, sex, level of education, country of residence, living status, history of CVD or diabetes, current smoking, physical activity, hypertension, dyslipidemia, obesity, intention to make lifestyle changes, cognition, depressive symptoms, anxiety, chronic condition self-management, physical performance, computer use during the 4 weeks before baseline, and diet. Table S1 in Multimedia Appendix 1 provides further details.

### Statistical Analyses

Baseline characteristics were described using means (SD), medians (IQR), or numbers (%) and were compared among low, moderate, and high engagers using 1-way ANOVA or Kruskal-Wallis tests for continuous variables and chi-square tests for categorical variables.

Baseline predictors of overall engagement (categorized as low, moderate, or high) were assessed using a multivariate generalized ordered logit (partial proportional odds) model using the Stata *gologit2* command (StataCorp LP) [35]. In this model, the proportional odds assumption (ie, that the relationship between each pair of outcome categories was the same) was assessed using Wald tests for each predictor variable. If the assumption held, only 1 coefficient was calculated for the predictor variable, as in standard ordinal logistic regression. If it was violated, separate coefficients were calculated for the comparison of low versus moderate and high engagement categories and for low and moderate versus high engagement categories. The initial multivariate model included all variables associated with engagement in bivariate models at the .2 significance level, and the final model was determined using a manual backward stepwise selection procedure (sequentially eliminating variables with a *P*>.05).

Baseline factors associated with the first occurrence of nonuse attrition were examined using multivariate Cox proportional hazards models. As the proportional hazards assumption (verified using Schoenfeld residuals) was not met in the initial analysis, the follow-up period was split, following visual inspection of Kaplan-Meier survival curves, into early (ie, months 0-2) and late (ie, month 3 onward) periods, and analyses were run separately for each period. Variable selection was performed as for the generalized ordered logit model.

Finally, 18-month changes in the HATICE primary and secondary outcome variables were compared between the control group and the 3 engagement categories in the intervention group using linear regression models, adjusted for baseline variables associated with engagement, age, education, physical status, and smoking. Models were further adjusted for the baseline score of the outcome of interest if it differed significantly among engagement groups.

All analyses were exploratory and performed using Stata (version 14.1).

## Results

### Description of Engagement

The median number of logins per participant in the intervention group (N=1389) during the 18-month follow-up period was 29 (IQR 16-48; range 0-700; Figure S1A in Multimedia Appendix 1). In comparison, the median number of logins to the static platform in the control group (N=1335) was 12 (IQR 9-16).

Of the 1389, intervention group participants, 1194 (85.96%) sent at least one message to their coach during the 18-month study period (Figure S1B in Multimedia Appendix 1), and the median (IQR) number of messages sent was 6 (2-10). The median (IQR) number of goals set was 1 (1-2), and of the 1389 participants, 151 (10.87%) did not set any goals, and 560 (40.32%) set 2 or more goals (Figure S1C in Multimedia Appendix 1). Weight was the health factor most commonly targeted by goals, followed by physical activity, and nutrition (Figure S1D in Multimedia Appendix 1). Participants were most likely to read advice and education pages for cholesterol, blood pressure, and diabetes (Figure S1E in Multimedia Appendix 1). Physical activity measurements (min/week, subjectively reported) were the most frequent type of monitoring data entered, followed by weight and blood pressure measurements (Figure S1F in Multimedia Appendix 1). Additional descriptive engagement data are provided in Table S2 in Multimedia Appendix 1.

The median (IQR) composite engagement score was 5 (3-6), and of the 1389 participants, 208 (14.97%) were classified as having low engagement with the platform, 681 (49.03%) moderate engagement, and 500 (36%) high engagement (Table S3 in Multimedia Appendix 1). All components of platform use significantly increased across the three categories (and tertiles; Table S4 in Multimedia Appendix 1).

### Predictors of Engagement

At baseline, participants in the low engagement category were younger, had a lower level of education, had poorer cognitive and physical performance, and had more depressive symptoms than those who engaged more. They were also more often from the Netherlands, more likely to be smokers, and less likely to have used a computer in the preceding 4 weeks or be planning or already acting on lifestyle change (Table 1).

In the multivariate analysis (Table 2), increasing engagement was independently predicted by country of residence, having short-term (ie, within the next month) plans for lifestyle change or acting on it for more than 6 months, and regular computer use at baseline. Furthermore, compared with those in the low engagement category, participants in the moderate and high engagement categories had better baseline cognitive performance, and compared with those in the low and moderate categories, those in the high engagement category were more likely to be women.

**Table 1:** HATICE^a^ participants’ (intervention group) baseline characteristics by overall engagement during the trial.

		Low engagement (N=208)	Moderate engagement (N=681)	High engagement (N=500)	*P* value	
Age (years), median (IQR)		69.6 (67.5-73.9)	69.5 (67.3-72.8)	69.0 (67.0-72.4)	.05	
Men, n (%)		102 (49)	385 (56.5)	244 (48.8)	.02	
**Education^b^, n (%)**						.02
	Low	82 (39.4)	201 (29.5)	134 (26.8)		
	Medium	58 (27.9)	206 (30.3)	159 (31.8)		
	High	68 (32.7)	274 (40.2)	207 (41.4)		
**Country, n (%)**						.006
	Netherlands	121 (58.2)	402 (59.0)	244 (48.8)		
	France	22 (10.6)	86 (12.6)	69 (13.8)		
	Finland	65 (31.3)	193 (28.3)	187 (37.4)		
Living with partner, n (%)		146 (70.2)	500 (73.4)	361 (72.2)	.65	
Cognitive *z* score^c^, mean (SD)		−0.14 (0.69)	0.03 (0.58)	0.00 (0.08)	.003	
SPPB^d,e^<10, n (%)		47 (22.6)	93 (13.7)	78 (15.6)	.008	
Depressive symptoms^f^, n (%)		27 (13)	54 (7.9)	39 (7.8)	.05	
HADS^g^ anxiety score^h^, median (IQR)		4 (2-6)	4 (2-6)	4 (2-6)	.16	
History of CVD^i^, n (%)		60 (28.9)	210 (31)	154 (30.9)	.83	
Diabetes, n (%)		41 (19.7)	154 (22.7)	101 (20.2)	.50	
Hypertension, n (%)		170 (82.9)	557 (83.5)	409 (83.3)	.98	
Dyslipidemia, n (%)		201 (97.1)	653 (96)	480 (96.6)	.74	
Currently smoking, n (%)		20 (12.1)	47 (7.5)	29 (5.9)	.03	
Physically active^j^, n (%)		128 (61.5)	452 (66.5)	334 (66.8)	.36	
Obese, n (%)		82 (39.4)	262 (38.5)	185 (37)	.79	
MEDAS^k^ score^l^, mean (SD)		5.8 (2.0)	6.2 (2.0)	6.0 (1.9)	.10	
PIH^m^ score^n^, median (IQR)		87 (79-91)	87 (81-91)	86 (81-91)	.53	
**Trying to change lifestyle? n (%)**						.001
	No plans	25 (12)	45 (6.6)	30 (6)		
	Long-term plans	27 (13)	78 (11.5)	40 (8)		
	Short-term plans	35 (16.8)	82 (12)	88 (17.6)		
	Short-term acting	39 (18.8)	120 (17.6)	92 (18.4)		
	Long-term acting	82 (39.4)	356 (52.3)	250 (50)		
**Computer use in the last 4 weeks, n (%)**						<.001
	No	22 (11)	18 (3)	3 (1)		
	Yes, <7 hours/week	113 (55)	395 (58)	276 (55)		
	Yes, ≥7 hours/week	72 (35)	267 (39)	221 (44)		

^a^HATICE: Healthy Ageing Through Internet Counselling in the Elderly.

^b^Low, medium, and high education levels correspond to basic, postsecondary nontertiary, and tertiary levels, respectively.

^c^Cognitive *z* score indicates average *z* scores of the Mini Mental Status Examination, Category Fluency, Stroop Color-Word Test, and Rey Auditory Verbal Learning Test.

^d^SPPB: Short Physical Performance Battery.

^e^Range 0-12 points, where higher scores indicate better performance.

^f^Geriatric Depression Scale–15 score ≤5.

^g^HADS: Hospital Anxiety and Depression Scale.

^h^Range 0-21, where higher scores indicate increasing symptoms of anxiety.

^i^CVD: cardiovascular disease.

^j^Defined as meeting the World Health Organization guidelines of ≥150 minutes’ moderate-intensity or ≥75 minutes’ vigorous-intensity physical activity per week.

^k^MEDAS: Mediterranean Diet Adherence Screener.

^l^Range 0-14, where higher scores indicate higher adherence to Mediterranean diet.

^m^PIH: Partners in Health.

^n^Range 0-96, where higher scores indicate better chronic disease self-management.

**Table 2:** Final multivariate^a^ generalized ordered logistic regression model showing factors significantly associated with increasing overall engagement during follow-up (categorized as low, moderate, or high platform engagement; N=1238).

				OR^b^ (95% CI)		*P* value^c^
**Variables meeting the proportional odds assumption^d^**						
	**Country**					*.02*
		Netherlands (ref^e^)	1		N/A^f^	
		France	1.41 (0.98-2.02)		.07	
		Finland	1.55 (1.16-2.06)		.003	
	**Trying to change lifestyle?**					*.002*
		No plans (ref)	1		N/A	
		Long-term plans	1.20 (0.70-2.07)		.51	
		Short-term plans	2.25 (1.33-3.80)		.002	
		Short-term acting	1.51 (0.92-2.50)		.11	
		Long-term acting	2.02 (1.26-3.25)		.004	
	**Computer use in last 4 weeks before baseline visit**					*<.001*
		None (ref)	1		N/A	
		<7 hours/week	5.39 (2.66-10.95)		<.001	
		≥7 hours/week	6.58 (3.21-13.49)		<.001	
**Variables not meeting the proportional odds assumption^g^**						
	**Low engagement (ref)** **vs moderate and high engagement**					
		Sex (male)	1.20 (0.84-1.72)		.31	
		Cognitive *z* score	1.67 (1.26-2.21)		<.001	
	**Low and moderate engagement (ref) vs high engagement**					
		Sex (male)	0.77 (0.60-0.98)		.03	
		Cognitive *z* score	0.99 (0.81-1.22)		.95	

^a^The following baseline variables were included in the initial multivariate model but did not remain significantly associated with engagement following a backward stepwise selection procedure: age, education, current smoking, physical status (Short Physical Performance Battery), depressive symptoms (Geriatric Depression Scale), anxiety (Hospital Anxiety and Depression Scale), and nutrition score.

^b^OR: odds ratio.

^c^*P* values in italics are overall Wald tests for categorical variables.

^d^For independent variables meeting the proportional odds assumption, the relationship between each pair of outcome categories (ie, moderate and high engagement vs low engagement and high engagement vs low and moderate engagement) is the same; therefore, only 1 OR is calculated per variable.

^e^ref: reference.

^f^N/A: not applicable.

^g^For independent variables not meeting the proportional odds assumption, separate ORs are calculated between each pair of outcome categories.

### Changes Over Time in Engagement and Its Associated Factors

**Figure 2:**
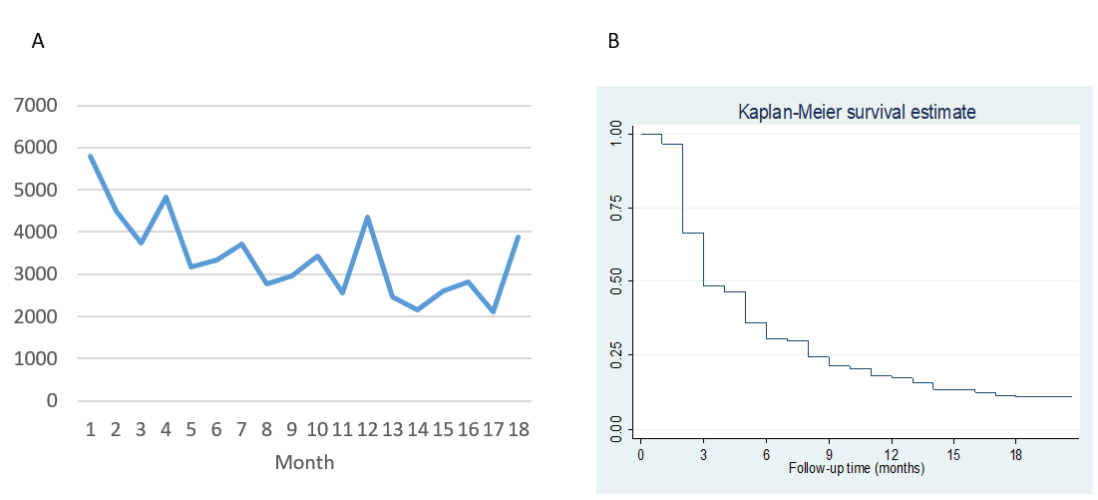
Changes in engagement over time in the intervention group: (A) total number of logins per month in the intervention group and (B) time to nonuse attrition (ie, no login during the previous month).

As shown in Figure 2A, intervention use (measured using logins) declined over time. The roughly (reverse) sigmoidal Kaplan-Meier nonuse attrition curve (Figure 2B) shows a sharp decrease in the proportion of participants logging in at least once during the previous month from the end of month 2 onward. In a sensitivity analysis using a 6-week nonuse attrition definition, the curve was similar, although slightly elongated (Figure S2 in Multimedia Appendix 1). The median time to the first occurrence of nonuse attrition was 3 months. Of the 1389 participants in the intervention group, 465 (33.48%) demonstrated early nonuse attrition (during months 1-2), 747 (53.78%) demonstrated late nonuse attrition (between 2 and 18 months), and 145 (10.44%) were highly consistent platform users, logging in at least once every month for the entire follow-up period (32/1389, 2.30% logged in every month, during which they participated in the trial but dropped out before the end of follow-up). The highly consistent users demonstrated significantly higher engagement with all parts of the platform than the other 2 groups, but even in the early nonuse attrition group, 24.30% (113/465) logged in to the platform at least once a month for at least 12 of the 18 months of follow-up (Table S5 in Multimedia Appendix 1).

Not using a computer regularly before baseline and a lower baseline chronic condition self-management score predicted early nonuse attrition, whereas living in the Netherlands and acting on lifestyle change for <6 months at baseline predicted late nonuse attrition (Table 3).

**Table 3:** Baseline factors associated with early (model 1) and late (model 2) nonuse attrition.

				HR^a^		95% CI		*P* value
**Model 1: early nonuse attrition^b^ (N=1351; 448 events)**								
	**Computer use in the last 4 weeks**							
		None (ref^c^)	1		N/A^d^		N/A	
		<7 hours/week	0.46		0.31-0.69		<.001	
		≥7 hours/week	0.44		0.29-0.66		<.001	
	Partners in Health score (points)^e^			0.99		0.98-1.00		.03
**Model 2: late nonuse attrition^f^ (N=848; 693 events)**								
	**Country**							
		Netherlands (ref)	1		N/A		N/A	
		France	0.66		0.51-0.84		.001	
		Finland	0.57		0.47-0.69		<.001	
	**Trying to change lifestyle?**							
		No plans to change lifestyle (ref)	1		N/A		N/A	
		Long-term plans to change lifestyle	0.96		0.66-1.40		.83	
		Short-term plans to change lifestyle	1.31		0.96-1.78		.09	
		Short-term acting on lifestyle change	1.49		1.15-1.93		.002	
		Long-term acting on lifestyle change	1.15		0.91-1.45		.23	

^a^HR: hazard ratio.

^b^The first instance of nonuse attrition during months 1 to 2. The following baseline variables were included in the initial multivariate model but did not remain significantly associated with early nonuse attrition following a backward stepwise selection procedure: education, history of cardiovascular disease, history of diabetes, history of hypertension, Geriatric Depression Scale score, and verbal fluency score.

^c^ref: reference.

^d^N/A: not applicable.

^e^Higher scores indicate better chronic disease self-management.

^f^The first instance of nonuse attrition from month 3 onward. The analysis included individuals who had not already undergone an episode of nonuse attrition during the first 2 months. The following variables were included in the initial multivariate model but did not remain significantly associated with late nonuse attrition following a backward stepwise selection procedure: education, current smoking, obesity, age, Mini Mental Status Examination score, verbal fluency score, Stroop score, Rey Auditory Verbal Learning Test recall score, Short Physical Performance Battery score, Partners in Health score, and Mediterranean Diet Adherence Screener nutrition score.

### Association Between Engagement and Intervention Outcomes

**Figure 3:**
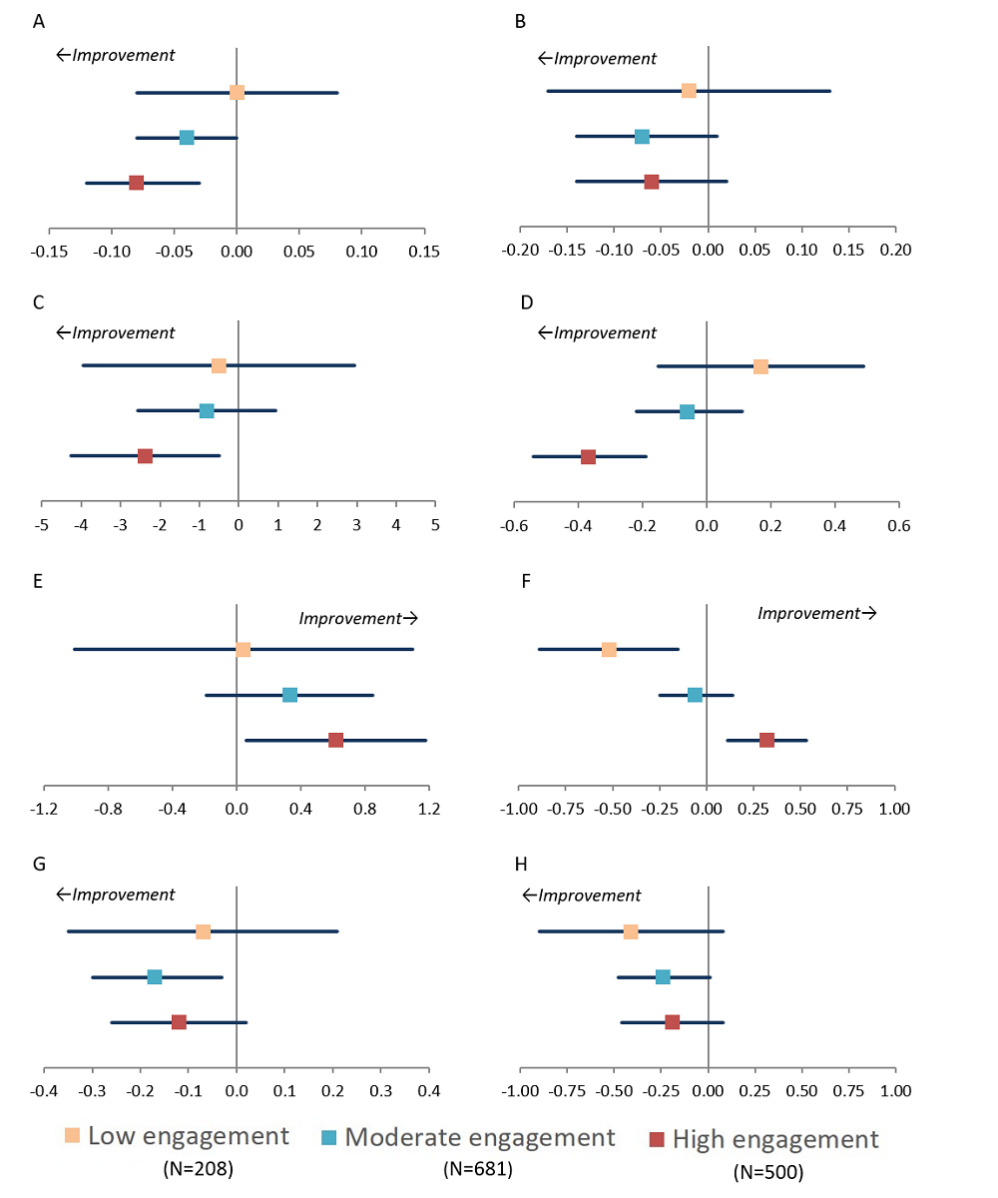
Adjusted mean difference in 18-month changes in outcome measures in low, moderate, and high engagement categories in the HATICE intervention group compared with control group: (A) HATICE composite z-score (BMI, LDL, and SBP), (B) LDL cholesterol (mmol/L), (C) SBP (mm Hg), (D) BMI (kg/m2), (E) moderate-intense physical activity (hours/week), (F) MEDAS score (range 0-14 points), (G) CAIDE dementia risk score (range 0-15 points), and (H) SCORE-OP (10-year CVD mortality risk). Point estimates are the mean difference in 18-month change compared with the control group. Bars are 95% CIs. Each model was adjusted for baseline age, sex, education, country, physical function, smoking, plans to make lifestyle changes, computer use, and cognition and for baseline score of the outcome of interest if it differed across engagement groups. The HATICE primary outcome measure was a composite score based on the average of 18-month changes in SBP, LDL cholesterol, and BMI z-scores. CAIDE: Cardiovascular Risk Factors, Aging, and Incidence of Dementia; CVD: cardiovascular disease; HATICE: Healthy Ageing Through Internet Counselling in the Elderly; LDL: low-density lipoprotein; MEDAS: Mediterranean Diet Adherence Screener; SCORE-OP: Systematic Coronary Risk Estimation–Older People; SBP: systolic blood pressure.

There was a significantly greater improvement in the HATICE primary outcome measure, comprising systolic blood pressure, BMI, and low-density lipoprotein cholesterol, over 18 months in the high engagement category than in the control group (adjusted mean difference −0.08, 95% CI −0.12 to −0.03; *P*=.001), with an indication of a dose-response effect (Figure 3 and Table S6 in Multimedia Appendix 1; overall *P* value across the 3 adherence groups=.005). Similarly, compared with those in the control group, there was a significantly greater decrease in systolic blood pressure and BMI and significantly less decline in physical activity and Mediterranean Diet Adherence Screener score (all indicating improvement of cardiovascular or dementia risk) in the high engagement category over 18 months (Figure 3 and Table S6 in Multimedia Appendix 1). The results were also numerically, if not significantly, in favor of greater improvement in the other outcome measures, except for Systematic Coronary Risk Estimation–Older People (SCORE-OP), in the high and moderate engagement groups. The results were comparable when the engagement scores were categorized into tertiles (Table S7 in Multimedia Appendix 1).

## Discussion

### Principal Findings

In an 18-month randomized trial in older adults, compared with those in the control group, those in the intervention group who engaged most with the eHealth intervention designed to encourage lifestyle changes showed significantly greater improvement in objectively and subjectively measured cardiovascular and dementia risk factors. Those with low engagement showed no difference compared with the control group. Participants who reported that they were already working on improving their lifestyle at baseline, or had short-term plans to do so, were more engaged with the intervention. Those who reported not using a computer in the month before baseline were extremely unlikely to engage, irrespective of their intentions regarding lifestyle change.

Although most intervention group participants engaged with the HATICE platform to some extent (eg, 1238/1389, 89.13% set at least one goal), some intervention components, notably the advice and education sections, were used less frequently than others. Interestingly, lifestyle factors (ie, weight loss and physical activity) were the most frequent targets for goal setting, but participants tended to read more advice and education materials when they set a goal relating to a clinical risk factor (ie, cholesterol, blood pressure, or diabetes), suggesting potential differences in engagement depending on underlying motivations.

### Strengths and Limitations

Our study provides comprehensive data concerning the engagement of older adults with a tailored digital health intervention over a relatively long period. To date, this population has received little attention in this field. We used data from a large international randomized controlled trial and used multiple objective measures of engagement combined into a composite indicator and a large range of validated predictor and health outcome variables. Our results can be interpreted alongside the qualitative research conducted within the same trial [19,21,36]. A limitation is that, given the difficulty in defining a suitable *dose* of eHealth interventions and a lack of consensus in the literature on how to measure engagement [10], our engagement indicator was arbitrarily defined after trial completion based on the distributions of the different metrics. However, the definition was chosen before conducting any of the comparative analyses presented here, and it adequately captured differences in engagement. Nonetheless, although some participants demonstrated very high levels of engagement with the eHealth intervention, this subsample was very small. Therefore, we could not specifically study the associations between this very high level of engagement and study outcomes. In addition, our participants, who had at least basic computer literacy (due to study eligibility criteria) and had consented to participate in an eHealth behavior change intervention trial, are not representative of the general older population, in which disparities in engagement would likely be greater. However, the multinational context and use of various recruitment methods have increased the diversity of our population.

### Comparison With Previous Work

As in younger populations, engagement declined over time in our trial, with a typical sigmoidal pattern of nonuse attrition indicating a *curiosity plateau* followed by a *rejection phase* [8]. Similarly, although older adults were less likely to participate in a web-based chronic disease self-management intervention trial than were younger adults, those who did participate engaged with the intervention in a manner similar to the younger participants [13]. Sustaining engagement may be vital for the long-term effectiveness of digital health interventions, as efficacy declines over time [6]. Automated reminders, and in particular human support, may increase engagement. Adherence to supervised lifestyle interventions appears greater than that to unsupervised interventions in older populations [37], and qualitative research with the HATICE participants underlined the motivational role of the coaches in this trial [36]. Participants also reported that user-friendliness, notably in terms of the attractiveness of the platform, and technical difficulties (eg, login problems) also influenced their engagement with the platform [36]. Integrating digital health interventions into primary care might enhance sustainability and provide additional motivation to older individuals not yet considering lifestyle changes or with reticence regarding such programs [22,36].

Similar to a Finnish computerized cognitive training intervention for older adults [38], and as mentioned in our qualitative work [36], previous level of computer use was the strongest predictor of engagement with our web-based intervention, even in our more contemporary population, in which basic computer literacy was an inclusion criterion. The notion of computer use may reflect both computer literacy and computer access (or quality of access) and is likely to be an indicator of inequalities in access to the eHealth intervention. Indeed, compared with HATICE participants who reported regular use at baseline (N=1344), those who did not regularly use a computer (N=43) were significantly more likely to be older and women and to have a lower level of cognition and education (data not shown). Moreover, Dutch participants were less likely to be regular computer users at baseline and engaged less with the platform than those in France and Finland. This could reflect cultural differences in attitudes toward behavior change, prevention, and research participation [19,21]. Furthermore, self-selection due to recruitment methods may have led the French and Finnish samples to be biased toward more motivated and health-focused individuals. Dutch participants, who were recruited via their general practitioners and were likely influenced by medical authority [19], appeared to be more representative of the general population, notably in terms of education level [17]. Participants’ intentions to make lifestyle changes were also associated with platform engagement, and better self-management of chronic conditions was associated with a lower risk of early nonuse attrition. Both relate to self-efficacy, an established predictor of adherence to lifestyle interventions [39].

Higher engagement with the HATICE intervention platform was associated with more favorable changes in the trial’s main cardiovascular and dementia risk outcome measures, with evidence of a dose-response effect. Similar results have been reported for eHealth interventions targeting various health conditions or behaviors in younger populations [10], but the mechanisms (or *mediators*, eg, increased knowledge, motivation, self-efficacy, or affect management) underlying such relationships are not well understood [24,25]. Furthermore, we assessed engagement using an aggregate indicator, suggested to be the most useful measure of engagement [10], but it is important to understand whether measures of frequency (eg, logins) and intensity (eg, number of messages sent or amount of advice and education read) of engagement and passive (eg, reading advice and education) and active (eg, entering measurements and sending messages) platform use all influenced outcomes similarly. Not all forms of engagement with eHealth interventions are necessarily associated with outcomes [29], and the frequency of engagement may be more associated with physical health outcomes [25], whereas intensity is more associated with psychological health outcomes [10]. In addition, it is important to understand whether intervention use is a valid indicator of engagement in behavior change [24] and, if so, through which mechanisms. Finally, engagement with eHealth interventions may encompass more than just objective measures of use, and factors such as interest, enjoyment, and attention may also play a role [24,40].

### Conclusions

Engaging older people in an eHealth lifestyle self-management intervention is feasible, and greater engagement is associated with greater improvement in biological and behavioral dementia and cardiovascular risk factors. Further work is required to determine more specifically the strength or type of engagement with such interventions required to obtain a meaningful impact on health outcomes and how best to sustain engagement over time. Our results also suggest disparities in engagement, which, given biases in trial participation, are likely to be accentuated in real-world settings. Older adults with limited computer experience, poorer cognition, and no concrete plans for lifestyle change may require extra support to reach a level of engagement with digital lifestyle interventions that is sufficient to bring about health benefits or require access to alternative methods of intervention delivery to mitigate potential health inequalities that could be associated with the widespread roll-out of digital health interventions in older populations.

## Appendix

Multimedia Appendix 1 Supplementary tables and figures.

## Abbreviations

aOR: adjusted odds ratio
CVD: cardiovascular disease
HATICE: Healthy Ageing Through Internet Counselling in the Elderly

## Data Availability

All individual participant data collected during the trial, after deidentification, and study documents, including the study protocol, will be available to academic researchers who provide a methodologically sound proposal, approved by the Healthy Ageing Through Internet Counselling in the Elderly steering group, and agree to collaborate on the analysis with at least one member of the Healthy Ageing Through Internet Counselling in the Elderly study team. Enquiries or proposals should be directed to MHB.
