# Supplementary material for: Factors Predicting Engagement of Older Adults With a Coach-Supported eHealth Intervention Promoting Lifestyle Change and Associations Between Engagement and Changes in Cardiovascular and Dementia Risk: Secondary Analysis of an 18-Month Multinational Randomized Controlled Trial
Source: J Med Internet Res. 2022 May 9;24(5):e32006. doi: 10.2196/32006 (PMC9127655; doi:10.2196/32006)
Supplement: Multimedia Appendix 1 [file jmir_v24i5e32006_app1.docx]

# Multimedia Appendix

**Supplementary Figure 1.** Summary of HATICE platform use during follow-up (intervention group only)

**
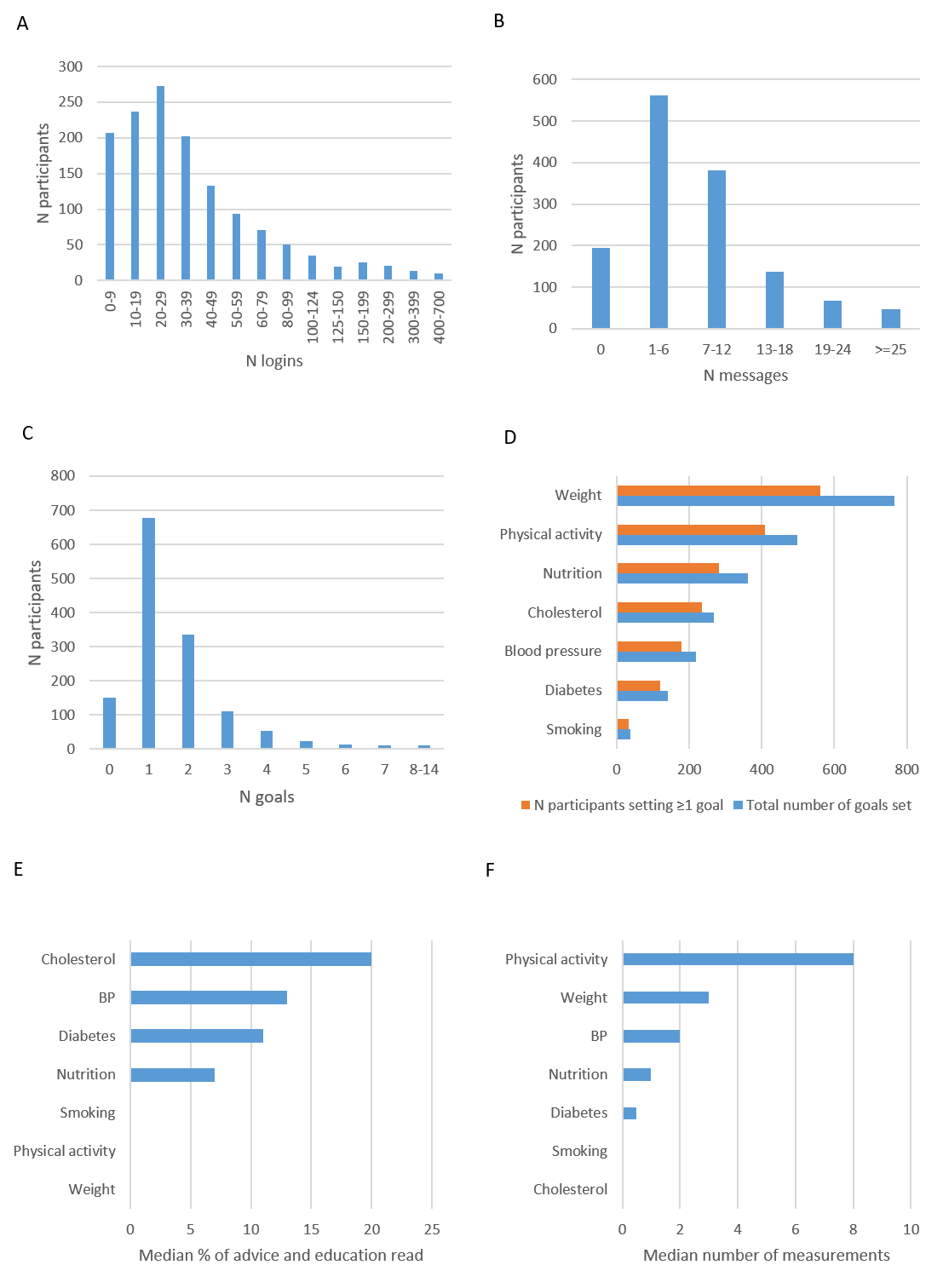
**

(A) total number of login per participant, (B) total number of messages sent per participant, (C) total number of goals set per participant, (D) goal setting by health factor, (E) median percentage of advice and education read among participants having set a goal for the health factor, and (F) median number of measurements among participants having set a goal for the health factor. HATICE: Healthy Ageing Through Internet Counselling in the Elderly.

**Supplementary Figure 2. Kaplan Meier curve using a 6-week definition of non-use attrition**

**Supplementary table 1: Details concerning measurement of baseline variables**

| **Variable** | **Instrument or criteria/categories** | **Reference** |
| --- | --- | --- |
| Level of education | - Basic - Post-secondary non-tertiary - Tertiary | N/A |
| Living status | - Living alone - Living with a partner (as a couple) - Living with somebody else | N/A |
| History of cardiovascular disease | Stroke/transient ischemic attack (TIA), myocardial infarction, angina pectoris, or peripheral arterial disease | N/A |
| History of diabetes | Diagnosis by specialist or general practitioner | N/A |
| Physical activity | Hours per week, as measured by the Community Health Program for Seniors Physical Activity Questionnaire (CHAMPS) | Stewart AL et al. Med Sci Sports Exerc. 2001;33(7):1126–1141. |
| Hypertension | Diagnosis by specialist or general practitioner; and/or use of anti-hypertensive drugs; and/or baseline BP: if < 80 years: ≥ 140/90 mmHg; if ≥ 80 years: Systolic BP ≥ 160 | N/A |
| Dyslipidaemia | Diagnosis by specialist or GP; and/or use of lipid-lowering drug; and/or baseline total cholesterol ≥ 5 mmol/L and/or LDL ≥ 2.5 mmol/L | N/A |
| Obesity | Body mass index (BMI)≥30 | N/A |
| Intention to make lifestyle changes | Are you already trying to change your lifestyle?   - No and not planning to do so within the next half year - No, but planning to within the next half year - No, but planning to within the next month - Yes, but acting on it for less than half a year - Yes and acting on it for more than half a year | N/A |
| Verbal fluency | Category fluency (animals) | Cardebat D et al. Acta Neurol Belg 1990; 90: 207–17 |
| Executive function | Stroop colour-word test | Stroop, J. R. *J. Exp. Psychol* (1935); 18: 643–662 |
| Memory | Rey Auditory Verbal Learning Test | Rey, A. Archives de Psychologie (1941); 28 : 286-340 |
| Global cognition | - MMSE - Composite z-score combining all 4 cognitive tests | Folstein MF et al. J Psychiatr Res 1975; 12: 189–98. |
| Depressive symptoms | Geriatric Depression Scale (GDS) | Yesavage JA, et al. J Psychiatr Res 1982; 17: 37–49. |
| Anxiety | Hospital Anxiety and Depression Scale (HADS) | Zigmond A.S., Snaith R.P. Acta Psychiatr. Scand., 1983, 67, 361-370. |
| Chronic condition self-management | Partners in Health (PIH) | Battersby MW et al. Aust J Prim Health. 2003;9:41–52 |
| Physical performance | Short Physical Performance Battery (SPPB) | Guralnik JM et al. J Gerontol A Biol Sci Med Sci 2000; 55: M221–31. |
| Computer use | CHAMPS (see above) item 6 | Stewart AL et al. Med Sci Sports Exerc. 2001;33(7):1126–1141. |
| Diet | Mediterranean diet adherence screener (MEDAS) | Schröder H, et al. J Nutr. 2011 Jun;141(6):1140-5. |

**Supplementary Table 2. Additional descriptive data concerning platform use in the intervention group of the HATICE trial**

|  | **N** | **%** |
| --- | --- | --- |
| Tertiles of logins  1 (0-21 logins)  2 (22-40 logins)  3 (41-700 logins) | 495  437  457 | 36  31  33 |
| Logged in ≥once a month | 143 | 10 |
| Logged in ≥12/18 months | 733 | 53 |
| Set ≥ 1 goal | 1238 | 89 |
| Set ≥ 2 goals | 560 | 40 |
| Read some A&E | 957 | 69 |
| Read ≥ 30% A&E for ≥1 health factor | 438 | 32 |
| ≥1 measurement/goal diary entry | 1071 | 77 |
| ≥5 measurements/goal diary entries | 751 | 54 |
| Tertiles of messages  1 (0-3 messages)  2 (4-8 messages)  3 (9-51 messages) | 493  435  461 | 35  31  33 |
| Sent ≥1 message | 1194 | 86 |

A&E: advice & education

**Supplementary Table 3. Distribution of composite engagement score**

| **Score** | **N** | **%** |
| --- | --- | --- |
| 0 | 25 | 1.80 |
| 1 | 66 | 4.75 |
| 2 | 117 | 8.42 |
| 3 | 162 | 11.66 |
| 4 | 244 | 17.57 |
| 5 | 275 | 19.80 |
| 6 | 266 | 19.15 |
| 7 | 234 | 16.85 |

The engagement score was calculated as the sum of:

- Logins (tertiles): 0/1/2 pts
- Goals (0/1/2+): 0/1/2 pts
- Messages (0 vs. ≥1): 0/1 pts
- Measurements (0 vs. ≥1): 0/1 pts
- Advice & education read (None vs. any): 0/1 pts

**Supplementary Table 4. Platform use by engagement score category or tertile**

|  | **Low engagement category**  **(0-2 points)**  **(N=208)** | **Moderate engagement category**  **(3-5 points)**  **(N=681)** | **High engagement category**  **(6-7 points)**  **(N=500)** | ***P* ^a^** | **Tertile 1**  **(0-4 points)**  **(N=370)** | **Tertile 2**  **(4-5 points)**  **(N=519)** | **Tertile 3**  **(6-7 points)**  **(N=500)** | ***P* ^a^** |
| --- | --- | --- | --- | --- | --- | --- | --- | --- |
| Logins: median (IQR) | 6 (2-12) | 23 (16-33) | 55 (41-91) | <.001 | 10 (4-17) | 26 (20-35) | 55 (41-91) | <.001 |
| Logged in ≥12/18 months: N(%) | 11 (5%) | 283 (42%) | 439 (88%) | <.001 | 34 (9%) | 260 (50%) | 439 (88%) | <.001 |
| Goals set: median (IQR) | 1 (0-1) | 1 (1-1) | 2 (2-3) | <.001 | 1 (0-1) | 1 (1-2) | 2 (2-3) | <.001 |
| Messages: median (IQR) | 0 (0-1) | 5 (2-8) | 10 (6-16) | <.001 | 1 (0-3) | 6 (3-9) | 10 (6-16) | <.001 |
| Read ≥30%A&E for ≥1 health factor: N(%) | 6 (3%) | 179 (27%) | 253 (51%) | <.001 | 32 (9%) | 153 (29%) | 253 (51%) | <.001 |
| ≥5 measurements/diary entries: N(%) | 20 (10%) | 321 (47%) | 410 (82%) | <.001 | 63 (17%) | 278 (54%) | 410 (82%) | <.001 |

A&E: advice & education; **^a^** Kruskall-Wallis tests for continuous variables; chi² tests for categorical variables

**Supplementary Table 5. Platform use by non-use attrition category**

|  | Early non-use attrition (N=465) | Late non-use attrition (N=747) | Highly consistent users  (N=145) | *P* **^a^** |
| --- | --- | --- | --- | --- |
| Logins: median (IQR) | 16 (7-26) | 33 (22-46) | 124 (88-205) | <.001 |
| Logged in ≥12/18 months: N(%) | 113 (24%) | 470 (63%) | 145 (100%) | <.001 |
| Goals set: median (IQR) | 1 (1-2) | 1 (1-2) | 2 (1-3) | <.001 |
| Messages: median (IQR) | 2 (0-7) | 7 (3-11) | 13 (8-20) | <.001 |
| Read ≥30%A&E for ≥1 health factor: N(%) | 94 (20%) | 257 (34%) | 78 (54%) | <.001 |
| ≥1 measurement/goal diary entry: N(%) | 285 (61%) | 622 (83%) | 143 (98%) | <.001 |

A&E: advice & education; **^a^** Kruskall-Wallis tests for continuous variables; chi² tests for categorical variables

**Supplementary Table 6. 18-month changes in outcome measures by engagement score, compared to the control group**

|  | **(Unadjusted) Mean (SD) 18m change** | | | | **Adjusted^a^ difference (95%CI) in 18m change, compared to control group**  ***[P-value]*** | | |
| --- | --- | --- | --- | --- | --- | --- | --- |
|  | **Control group**  **(N=1335)** | **Intervention group, low engagement**  **(N=208)** | **Intervention group, moderate engagement**  **(N=681)** | **Intervention group, high engagement**  **(N=500)** | **Intervention group, low engagement**  **(N=208)** | **Intervention group, moderate engagement**  **(N=681)** | **Intervention group, high engagement**  **(N=500)** |
| HATICE composite primary outcome^b^ | -0.04 (0.44) | -0.03 (0.42) | -0.08 (0.44) | -0.12 (0.47) | 0.00 (-0.08, 0.08)  *[P=.95]* | -0.04 (-0.08, 0.00)  *[P=.08]* | -0.08 (-0.12, -0.03)  *[P=.001]* |
| LDL cholesterol (mmol/l) | -0.07 (0.73) | -0.06 (0.70) | -0.13 (0.77) | -0.12 (0.80) | -0.02 (-0.17, 0.13)  *[P=.79]* | -0.07 (-0.14, 0.01)  *[P=.08]* | -0.06 (-0.14, 0.02)  *[P=.16]* |
| SBP (mmHg) | -0.67 (17.14) | -0.57 (17.80) | -1.41 (17.7) | -2.58 (17.66) | -0.50 (-3.95, 2.95)  *[P=.77]* | -0.82 (-2.57, 0.93)  *[P=.36]* | -2.37 (-4.25, -0.49)  *[P=.01]* |
| BMI (kg/ m2) | -0.06 (1.64) | -0.02 (1.28) | -0.12 (1.81) | -0.42 (1.43) | 0.17 (-0.15, 0.49)  *[P=.30]* | -0.06 (-0.22, 0.11)  *[P=.51]* | -0.37 (-0.54, -0.19)  *[P<.001]* |
| Moderate-intense physical activity (hours/wk) | -0.62 (0.15^a^) | -0.64 (0.47^a^) | -0.24 (0.21^a^) | -0.05 (0.24^a^) | 0.04 (-1.01, 1.10)  *[P=.94]* | 0.33 (-0.19, 0.85)  *[P=.22]* | 0.62 (0.06, 1.18)  *[P=.03]* |
| MEDAS score (range 0-14 points) | -0.04 (0.06^a^) | -0.77 (0.16^a^) | -0.14 (0.08^a^) | 0.32 (0.09^a^) | *-0.52 (-0.89, -0.15)*  *[P=.006]* | -0.06 (-0.25, 0.14)  *[P=.58]* | 0.32 (0.11, 0.53)  *[P=.003]* |
| CAIDE dementia risk score (range 0-15 points) | -0.04 (1.5) | -0.10 (1.4) | -0.22 (1.5) | -0.18 (1.5) | -0.07 (-0.35, 0.21)  *[P=.63]* | -0.17 (-0.30, -0.03)  *[P=.01]* | -0.12 (-0.26, 0.02)  *[P=.10]* |
| SCORE OP (% 10 year CVD risk) | -0.14 (2.5) | -0.59 (5.1) | -0.32 (2.3) | -0.24 (2.3) | -0.41 (-0.90, 0.08)  *[P=.10]* | -0.24 (-0.48, 0.01)  *[P=.06]* | -0.19 (-0.46, 0.08)  *[P=.16]* |

^a^ each model was adjusted for baseline age, sex, education, country, physical function, smoking, plans to make lifestyle changes, computer use, and cognition, and additionally for baseline score of the outcome of interest if it differed across engagement groups;  ^b^ average z-score of systolic blood pressure, LDL cholesterol and BMI

BMI: body mass index; CAIDE: Cardiovascular Risk Factors, Ageing and Incidence of Dementia; LDL: low density lipoprotein; MEDAS: Mediterranean Diet Adherence Screener; SCORE OP: Systematic Coronary Risk Estimation-Older People; SBP: systolic blood pressure

**Supplementary Table 7. Adjusted difference in 18-month changes in outcome measures by engagement score tertile, compared to the control group**

|  | **Adjusted^a^ difference (95%CI) in 18m change, compared to control group**  ***[p-value]*** | | |
| --- | --- | --- | --- |
|  | **Tertile 1**  **(0-4 points)**  **(N=370)** | **Tertile 2**  **(4-5 points)**  **(N=519)** | **Tertile 3**  **(6-7 points)**  **(N=500)** |
| HATICE composite primary outcome^b^ | 0.01 (-0.05, 0.07)  *[P=.76]* | -0.05 (-0.10, -0.01)  *[P=.02]* | -0.08 (-0.12, -0.03)  *[P=.001]* |
| LDL cholesterol (mmol/l) | -0.01 (-0.12, 0.10)  *[P=.84]* | -0.08 (-0.17, 0.00)  *[P=.04]* | -0.06 (-0.14, 0.02)  *[P=.16]* |
| SBP (mmHg) | -0.03 (-2.48, 2.43)  *[P=.08]* | -1.16 (-3.07, 0.74)  *[P=.23]* | -2.37 (-4.25, -0.49)  *[P=.01]* |
| BMI (kg/ m2) | 0.05 (-0.18, 0.28)  *[P=.69]* | -0.05 (-0.23, 0.12)  *[P=.56]* | -0.37 (-0.54, -0.19)  *[p<.001]* |
| Moderate-intense physical activity (hours/wk) | 0.28 (-0.47, 1.03)  *[P=.47]* | 0.29 (-0.28, 1.03)  *[P=.31]* | 0.62 (0.06, 1.18)  *[P=.03]* |
| MEDAS score (range 0-14 points) | -0.35 (-0.62, -0.08)  *[P=.01]* | -0.02 (-0.23, 0.19)  *[P=.87]* | 0.32 (0.11, 0.53)  *[P=.003]* |
| CAIDE dementia risk score (range 0-15 points) | -0.04 (-0.27, 0.19)  *[P=.73]* | -0.21 (-0.37, -0.04)  *[P=.02]* | -0.15 (-0.31, 0.02)  *[P=.08]* |
| SCORE OP (% 10 year CVD risk) | -0.17 (-0.52, 0.17)  *[P=.33]* | -0.31 (-0.58, -0.04)  *[P=.03]* | -0.19 (-0.46, 0.07)  *[P=.16]* |

^a^ each model was adjusted for baseline age, sex, education, country, physical function, smoking, plans to make lifestyle changes, computer use, and cognition, and additionally for baseline score of the outcome of interest if it differed across engagement groups;  ^b^ average z-score of systolic blood pressure, LDL cholesterol and BMI

BMI: body mass index; CAIDE: Cardiovascular Risk Factors, Ageing and Incidence of Dementia; LDL: low density lipoprotein; MEDAS: Mediterranean Diet Adherence Screener; SCORE OP: Systematic Coronary Risk Estimation-Older People; SBP: systolic blood pressure
